# Synergistic Regulation of Waste Cooking Oil Fractions for Asphalt Rejuvenation: Impact of Molecular Weight on Rheological Properties and Thermal Stability

**DOI:** 10.3390/ma19101924

**Published:** 2026-05-08

**Authors:** Rui Song, Shouqian Ni, Anqi Weng, Qunshan Ye, Gangping Jiang

**Affiliations:** 1 International College of Engineering, Changsha University of Science & Technology, Changsha 410114, China; 202327010226@csust.edu.cn; 2 School of Transportation, Changsha University of Science & Technology, Changsha 410114, China; 24101030053@csust.edu.cn (S.N.); 22201060254@stu.csust.edu.cn (A.W.);

**Keywords:** waste cooking oil, molecular weight distribution, component synergistic regulation, rejuvenated asphalt, rheological properties, MSCR

## Abstract

Owing to the pronounced compositional heterogeneity of waste cooking oil (WCO), WCO-rejuvenated asphalt often exhibits unstable performance. To improve the compositional controllability of WCO-based rejuvenators, WCO was fractionated according to molecular weight differences into three characteristic fractions: light, medium, and heavy components. Nine rejuvenator formulations with different component ratios were prepared to investigate the synergistic mechanism among WCO fractions with different molecular weights and to propose an optimal blending range. Thermal stability tests, dynamic shear rheometer (DSR) tests, multiple stress creep recovery (MSCR) tests, and bending beam rheometer (BBR) tests were conducted to evaluate the performance of the rejuvenators and rejuvenated asphalts. Gas chromatography (GC) and gel permeation chromatography (GPC) were further used to analyze the chemical composition and molecular-weight distribution. The results show that increasing the proportions of light and medium WCO components improves the low-temperature performance of rejuvenated asphalt; however, when the combined content of light and medium components exceeds 40%, the high-temperature performance is adversely affected. The heavy component improves the rutting factor, creep recovery capacity, and thermal oxidative aging resistance of rejuvenated asphalt, and the coefficient of determination between the long-term aging CAI and heavy-component content reaches 0.959. Thermal stability tests show that the mass loss rate of the nine rejuvenators after 1.5 h of heating ranges from 2.8% to 4.3%, with greater mass loss for formulations containing higher light-component contents. GPC results show that the Mn and Mw of R-4 (492 g/mol and 641 g/mol) are higher than those of R-8 (463 g/mol and 600 g/mol), indicating that a higher macromolecular fraction contributes to improved thermal stability. Considering high-temperature, low-temperature, and aging performance together, rejuvenated asphalt achieves the closest overall performance to the base binder when the heavy component is controlled at 50–60% and the medium component is approximately 30%.

## 1. Introduction

Asphalt pavements are susceptible to various forms of distress due to factors such as UV radiation, rainfall, and traffic loads. The design lifespan of asphalt pavements is typically 10 to 15 years; however, many pavements require significant repairs within 10 years of construction. During road maintenance, substantial amounts of reclaimed asphalt pavement (RAP) are produced [1,2,3]. Recycling RAP reduces pavement construction costs, lowers the consumption of virgin materials and energy, and substantially mitigates the environmental burdens associated with waste disposal. Therefore, it is a key strategy for sustainable pavement engineering and offers considerable potential for resource recovery [4,5]. Deploying recycling technologies for the large-scale circular utilization of RAP [6,7] is an urgent, essential strategy to advance green highway infrastructure, promote coordinated reductions in pollution and carbon emissions, and drive an economy-wide green transition [8,9].

Currently, both petroleum-based and bio-based rejuvenators are widely employed for the rejuvenation of aged asphalt. However, petroleum-based rejuvenators deplete non-renewable resources and demonstrate low regeneration efficiency, which impedes the sustainable development of highway transportation [10]. In contrast to petroleum-based rejuvenators, bio-oil derived from renewable resources, such as crop straw, wood chip, animal manure, and waste oil, can also regenerate aged asphalt [11]. Moreover, bio-rejuvenators enhance the resistance of aged asphalt mixtures to low-temperature cracking and increase their thermal stability, resulting in an efficient restoration of their performance [12,13,14].

Waste cooking oil (WCO) refers to used edible oil that is no longer suitable for reuse after cooking [15,16,17]. Improper disposal of WCO can cause negative environmental impacts. For example, direct discharge of WCO may cause local water eutrophication, stimulate the excessive growth of microorganisms, plankton, and algae, and disrupt the original ecological balance [18]. Large quantities of WCO are generated globally every year; therefore, the appropriate, rational, and safe treatment of WCO has attracted increasing attention [19,20].

Existing studies have confirmed the feasibility of using crude or esterified WCO as an asphalt rejuvenator. Zargar [21] found that adding 3–4% WCO brings the aged 40/50 grade asphalt’s physical state and rheological response back to values comparable to those of the original 80/100 grade asphalt. Asli [22] investigated the physical properties of WCO as a rejuvenator in reclaimed asphalt mixtures and recommended that up to 5% waste cooking oil be used without compromising the performance of the pavement mixture. Li [23] reported that the incorporation of waste engine oil and waste cooking oil into aged asphalt markedly reduces its stiffness and recovers satisfactory workability. Oldham [24] and his colleagues improved the water stability of asphalt by lowering the acid value of WCO through esterification with methanol. Recent research has also attempted to separate WCO into fractions with different molecular weights for asphalt rejuvenation. Zhang [25] divided WCO into small, medium, and macromolecular substances according to relative molecular mass and prepared corresponding rejuvenators for aged asphalt, indicating that the recovery effect of a single fraction is limited. Overall, previous studies have mainly focused on the rejuvenation effect of whole WCO or a single WCO-derived fraction, whereas the synergistic action of light, medium, and heavy molecular weight fractions and their systematic regulation of the rheological properties of rejuvenated asphalt remain insufficiently explored [26,27]. During asphalt aging, light maltene phases are lost, while more polar and higher-molecular-weight components accumulate, increasing binder stiffness and reducing molecular mobility. Therefore, an effective rejuvenator should not only soften aged asphalt, but also supplement maltene-rich phases and rebuild the colloidal balance between the maltene phase and asphaltene-rich domains. Compared with directly using crude WCO or a single fraction, the proposed blending strategy enables targeted performance regulation of aged asphalt.

This study categorizes WCO into three distinct components—light, medium, and heavy components—based on the differences in their physical properties. Nine rejuvenator formulations with different component ratios were investigated. Rheological tests were conducted to examine the impact of component type and blending ratio on the thermal stability of the rejuvenator and the rheological properties of rejuvenated asphalt. Additionally, gas chromatography (GC) and gel permeation chromatography (GPC) were employed to examine how compositional control influences the performance of the WCO rejuvenator. This study elucidates the impact of component type and ratio on the thermal stability of the rejuvenator and the rheological properties of rejuvenated asphalt, providing insights into the component design of WCO rejuvenators and their targeted repair mechanisms with aged asphalt.

## 2. Materials and Methods

### 2.1. Materials

#### 2.1.1. Base Asphalt

The base asphalt used was Grade 70 petroleum asphalt, produced by Hunan Baoli Asphalt Co., Ltd., Changsha, China. The detailed specifications are provided in Table 1.

#### 2.1.2. Waste Cooking Oil Components

**Figure 1:**
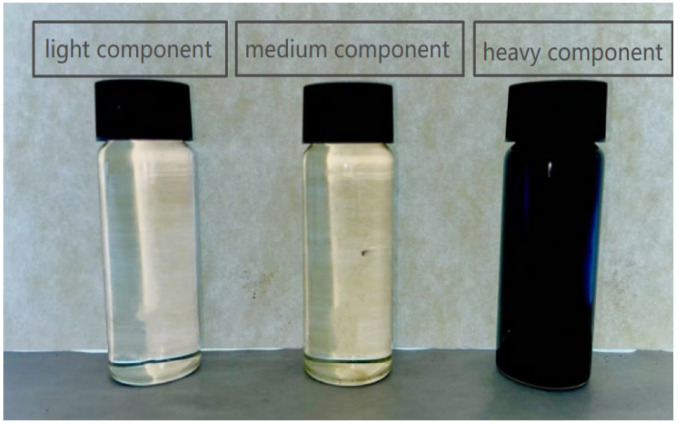
Components of WCO.

The waste cooking oil components were obtained from an energy technology company in Zhejiang, using local kitchen waste oils, gutter oils, and other discarded fats as raw materials. The oils underwent pretreatment processes, including impurity filtration, distillation for dehydration, and esterification for acid reduction. Finally, a distillation process was employed, where the temperature and pressure are controlled to obtain three components with different molecular weights: light, medium, and heavy components. Figure 1 shows the appearance of each component.

The light component mainly consists of methyl palmitate, with trace amounts of methyl myristate and methyl oleate. It has a relative molecular weight range of 200–300 and appears as a colorless, transparent liquid at room temperature. The medium component predominantly consists of methyl oleate, with a minor amount of methyl erucate. Its molecular weight ranges from 300 to 400, and it appears as a pale yellow liquid at room temperature. The heavy component is primarily composed of long-chain fatty acids, including stearic acid, arachidic acid, and lignoceric acid, with a molecular weight range of 500–1000. At room temperature, it typically appears as a dark brown, viscous oil with low fluidity. Table 2 presents the primary compositional profiles of the light, medium, and heavy components, and their performance indicators are summarized in Table 3.

### 2.2. Test Methods

#### 2.2.1. Preparation of Aged Asphalt

In accordance with ASTM D2872 [40], 35 g ± 0.5 g of 70# base asphalt was placed in a standard glass container and subjected to continuous rotational heating with air circulation at 163 °C ± 0.5 °C for 85 min. The asphalt was subsequently removed from the container to obtain the short-term aged asphalt. In accordance with ASTM D6521 [41], 50 g ± 0.5 g of the short-term aged asphalt sample was placed on an oven tray and heated until a smooth surface was achieved. The testing conditions were controlled at a temperature of 100 °C ± 0.5 °C, an air pressure of 2.1 MPa, and a duration of 20 h to prepare the long-term aged asphalt sample.

#### 2.2.2. Preparation of WCO Rejuvenator

To systematically investigate the synergistic regulation effects of the light, medium, and heavy fractions of waste cooking oil on the performance of the rejuvenator, nine WCO-based rejuvenator formulations with different component proportions were prepared in this study. The formulation matrix presented in Table 4 was developed using a comparative component-screening design, covering low, medium, and high concentration levels of each fraction within the practical formulation range defined by the separated WCO components. This design enables a systematic comparison of the relative contributions of the light, medium, and heavy fractions to the performance of the rejuvenator.

#### 2.2.3. Preparation Process of Recycled Asphalt

The WCO rejuvenator was first placed in an 80 °C oven for 30 min until it reached a fluid state. The mixture was then sheared at 500 rpm for 5 min using a high-speed shear device to ensure uniform mixing of the light, medium, and heavy components. The fluid rejuvenator was then mixed with 200 g of long-term aged asphalt in the molten state. The mixture was sheared using a high-speed shear device at 130 °C ± 5 °C and 2000 rpm for 25 min to produce recycled asphalt.

#### 2.2.4. Determination of the Thermal Stability of the WCO Rejuvenator

Rejuvenator formulations were prepared as specified in Table 4. For each formulation, mass, viscosity, and saponification value were determined after heating for 0.5, 1.0, and 1.5 h. Thermal stability of the WCO rejuvenator was evaluated using the following procedures: (1) Saponification value (SV): SV of the WCO rejuvenators was measured according to GB/T 5534-2008 [42]. (2) Dynamic viscosity at 60 °C: it was evaluated using the method described in GB/T 0619-2011 [43].

#### 2.2.5. Rheological Testing of Asphalt

(1) High-temperature rheological performance test: The base, aged, and rejuvenated asphalts were evaluated using an Anton Paar MCR 302 dynamic shear rheometer through the following tests: frequency scanning (temperature: 4, 16, 28, 40, and 52 °C; frequency: 0.016 Hz~16 Hz), temperature scanning (temperature: 40 °C~82 °C; angular frequency: 10 rad/s; strain level: 6%), and multiple stress creep recovery tests (1. Stresses: 0.1 kPa, cycles: 20; 2. Stresses: 3.2 kPa, cycles: 10).
(2) Low-temperature rheological performance test: In accordance with ASTM D6648 [44], a bending beam rheometer was used to measure creep stiffness *S* and the m-value (creep rate) for base, aged, and rejuvenated asphalt binders at −12, −18, and −24 °C.

#### 2.2.6. Gas Chromatography

Gas chromatography (GC) was performed on an Agilent J&W CP-Sil 88 capillary column. The injector was maintained at 270 °C in split mode (100:1). Nitrogen was used as the carrier gas at a constant flow of 0.70 mL·min^−1^. The oven temperature program was as follows: 100 °C for 13 min; ramp at 10 °C·min^−1^ to 180 °C and hold for 8 min; ramp at 1 °C·min^−1^ to 200 °C and hold for 20 min; ramp at 4 °C·min^−1^ to 230 °C and hold for 10.5 min. Detection employed a flame ionization detector (FID) at 280 °C with hydrogen at 400.7 mL·min^−1^, air at 4000.7 mL·min^−1^, and make-up gas at 250.7 mL·min^−1^.

#### 2.2.7. Gel Permeation Chromatography

A PL-GPC50 gel permeation chromatography (GPC) system was used to characterize the molecular weight distribution of rejuvenators R-4 and R-8. Tetrahydrofuran (THF, 2 mg/mL) was used as the organic solvent. A sample injection volume of 100 μL was used, and elution was carried out for 20 min at 40 °C.

#### 2.2.8. Repeatability and Data Presentation

All laboratory tests were conducted with at least three parallel measurements where applicable. The plotted data are reported as mean values, and the error bars in the revised figures represent the standard deviation.

## 3. Results and Discussion

### 3.1. Effect of Component Proportions on the Thermal Stability of the WCO Rejuvenator

#### 3.1.1. Mass Loss Rate

**Figure 2:**
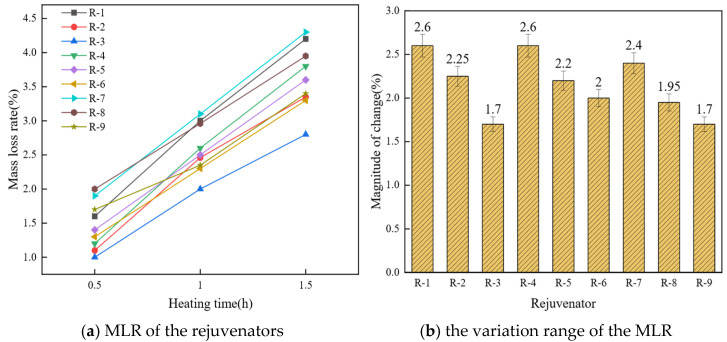
The effect of component ratio on the MLR of the rejuvenator.

The mass loss rate (MLR) test is a key metric for evaluating the thermal stability and durability of rejuvenated asphalt binders. It simulates high-temperature exposure and quantifies the percentage change in specimen mass over defined intervals. Rejuvenators with higher volatility exhibit greater post-heating mass loss, resulting in higher MLRs. Figure 2 presents the MLRs of the nine WCO rejuvenators and their ranges.

As shown in Figure 2a, rejuvenator R-8 exhibits the highest mass loss rate at 2% after 0.5 h of heating, followed by R-7, while R-3 shows the lowest mass loss rate at 1%. As the heating duration increases, the mass loss rates of all rejuvenators increase. After 1.5 h of heating, R-7 exhibits the highest mass loss rate (4.3%), followed by R-1 at 4.2%, while R-3 shows the lowest mass loss rate (2.8%). This phenomenon suggests that the initial mass loss rate of the rejuvenators is directly proportional to the content of light components. The boiling point of palmitic acid, a major light component, is lower than that of other fatty acids, such as eicosanoic acid and stearic acid, found in the rejuvenators [45]. Therefore, during the initial heating phase, R-8, with the highest proportion of light components, and R-3, with the lowest, exhibit opposite behaviors. Furthermore, an increase in light components decreases the activation energy of the bio-oil rejuvenators [46], leading to a decline in high-temperature performance. As shown in Figure 2b, the proportion of heavy components in R-1, R-2, and R-3 increases progressively, while the variation in the mass loss rate decreases. This indicates that the MLR of the rejuvenators is inversely correlated with the content of heavy components. Once the low-boiling light oils evaporate, the high-boiling heavy oils reduce the rate of mass loss increase. Therefore, increasing the heavy-component content enhances rejuvenator thermal stability. A higher initial MLR also implies that the rejuvenator may undergo compositional drift during hot-mix asphalt (HMA) production. Although the light fraction can provide short-term softening and viscosity reduction, rapid volatilization may lead to the loss of effective rejuvenating components during service and consequently reduce long-term cracking resistance.

#### 3.1.2. Dynamic Viscosity at 60 °C

**Figure 3:**
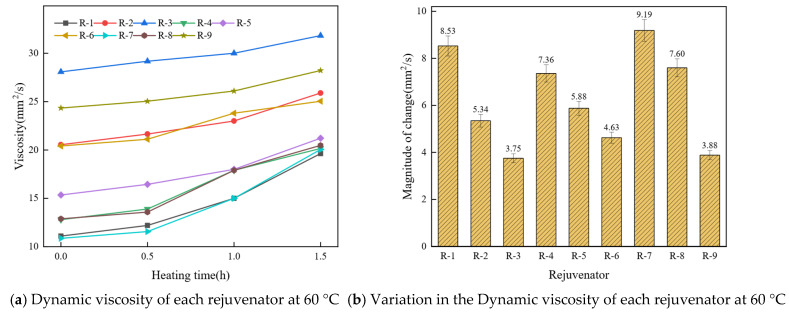
Influence of component proportions on the viscosity of the rejuvenator.

The dynamic viscosity at 60 °C is a key indicator of asphalt’s flowability, reflecting its resistance to flow at this temperature. A significant reduction in dynamic viscosity after using rejuvenators indicates that the rejuvenator effectively improves the asphalt’s aging properties. Figure 3 presents the dynamic viscosity and its variation range for the nine rejuvenator groups.

As shown in Figure 3a, the viscosity of all nine rejuvenator groups increases over time during heating. As shown in Figure 3b, the viscosity variation in R-1, R-4, R-7, and R-8 is considerably higher than that of the other five rejuvenator groups. During heating, substances with lower molecular weights continuously evaporate from the rejuvenators, leading to an increase in their average molecular weight and a corresponding rise in viscosity. Additionally, unsaturated fatty acids, such as oleic acid and linoleic acid, are more susceptible to oxidation and thermal polymerization at elevated temperatures [47], leading to an increase in viscosity as the oil becomes more viscous. Thus, the dynamic viscosity at 60 °C of rejuvenators improves as the proportion of light and medium components decreases.

#### 3.1.3. Saponification Value

**Figure 4:**
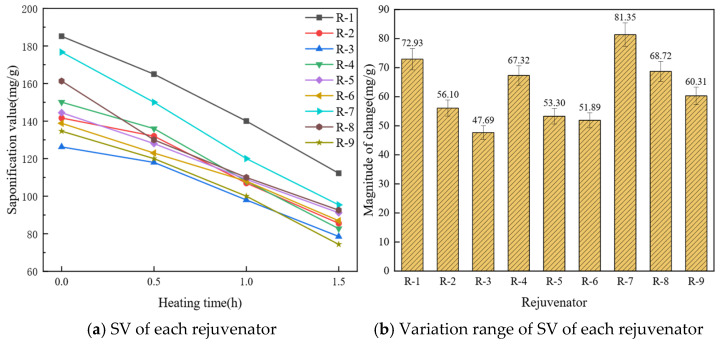
Influence of component proportions on rejuvenator saponification value.

The saponification value (SV) of a lipid sample primarily reflects the composition and abundance of esterified fatty acids. Because SV is inversely related to average molar mass [48], it can be used to reflect a sample’s average molar mass. In rejuvenators, an elevated SV coupled with chemically labile constituents indicates susceptibility to high-temperature reactions and consequent thermal instability. SV and its variation range for the nine rejuvenator groups are presented in Figure 4.

As shown in Figure 4a, SV of each rejuvenator decreases with increasing heating time, indicating an increase in their average molecular weight. During heating, low-molecular-weight oils continuously evaporate, while high-molecular-weight substances, which have better thermal stability, are retained. Consequently, SV of each rejuvenator decreases, whereas the average molecular weight increases. As shown in Figure 4b, R-3 exhibits the smallest change in SV, while R-7 shows the largest variation. This is because R-7 has the lowest proportion of heavy components and the highest proportion of light components among the nine rejuvenator groups. Consequently, increasing the proportion of heavy components improves the thermal stability of rejuvenators. When comparing R-7 to R-1, both have the same content of heavy components, but R-1 shows a smaller variation in SV. This is because R-1 contains 7.5% fewer light components than R-7, suggesting that the thermal stability of rejuvenators improves as the proportion of light components decreases.

### 3.2. Study on the Component Regulation Mechanism of WCO Rejuvenators

#### 3.2.1. Gas Chromatography (GC) Analysis

GC is an analytical technique that separates volatile and semi-volatile components in a sample by multiple distributions between the stationary phase and carrier gas. It characterizes the components qualitatively and semi-quantitatively using retention time and peak area. GC can analyze the chemical composition of volatile and semi-volatile organic components in asphalt, providing both qualitative and quantitative insights into the molecular-level chemical changes in asphalt and its rejuvenation system. Since the Flame Ionization Detector (FID) cannot detect substances with molecular weights exceeding 500, rejuvenators R-4 and R-8 were selected for the experiment. The selection rationale is as follows: R-8 has the highest proportion of light components, which are easier to monitor, while R-4 contains fewer heavy components and shows differences from R-8 in all three components. GC analysis was performed on R-4 and R-8, and their chemical compositions are provided in Table 5.

Table 4 shows that the light and medium components in R-4 constitute 8% and 36%, respectively, whereas in R-8, these proportions are 15.78% and 31.58%, respectively. Table 5 shows that the proportion of methyl palmitate in the light component of R-4 is 11.71%, whereas the proportion of methyl oleate in the medium component is 40.60%; In R-8, the proportion of methyl palmitate in the light component is 17.10%, while the proportion of methyl oleate in the medium component is 33.51%, with the remaining components classified as heavy components. Thus, the methyl palmitate content in R-8 is 5.39% higher than that in R-4, indicating that R-8 contains a higher proportion of light components but lower thermal stability than R-4. This suggests that thermal stability decreases as the content of light components increases.

#### 3.2.2. Gel Permeation Chromatography (GPC) Analysis

GPC is an analytical technique that separates samples by molecular volume using porous stationary phases under ideal swelling or non-adsorptive conditions. The elution volume is then converted to an apparent molecular weight distribution using a calibration curve. GPC is used to characterize the molecular composition of asphalt by determining its molecular weight distribution. The molecular weight distribution parameters for R-4 and R-8 are provided in Table 6.

Table 6 indicates that the weight-average molecular weight (M_w_) and number-average molecular weight (M_n_) of R-4 exceed those of R-8, implying that R-8 contains a greater proportion of small and medium-sized molecules. The Z-average molecular weight (M_Z_) is the cubic-weighted average of the molecular weight, and the concentration of larger molecules significantly affects this value. M_Z_ and the polydispersity index (PDI) of R-4 exceed those of R-8, suggesting that R-4 contains a higher proportion of large molecules. Moreover, R-4 exhibits greater thermal stability than R-8, implying that an increased concentration of heavy components contributes to the rejuvenator’s enhanced thermal stability. From the perspective of asphalt colloidal chemistry, the observed molecular weight differences also explain the rheological changes. The light and medium fractions mainly supplement maltene-like phases and promote diffusion within the aged asphalt colloid, thereby reducing rigidity and improving relaxation behavior. The heavy fraction provides larger and more strongly associated molecules, improves the solvation environment around asphaltene-rich domains, and enhances intermolecular associations within the rejuvenated asphalt colloid. When the light fraction is excessive, its effect shifts toward dilution of the asphaltene-rich structure, which lowers viscosity and improves low-temperature relaxation but weakens thermal stability and rutting resistance.

### 3.3. Rheological Performance Analysis

To facilitate the analysis of the effect of varying component ratios on the rheological properties of recycled asphalt, the proportions of light, medium, and heavy components in the nine composite WCO rejuvenators were calculated based on Table 4 and are presented in Table 7. The naming convention for the recycled asphalt is consistent with that in Table 7.

#### 3.3.1. High-Temperature Rheological Properties

**Figure 5:**
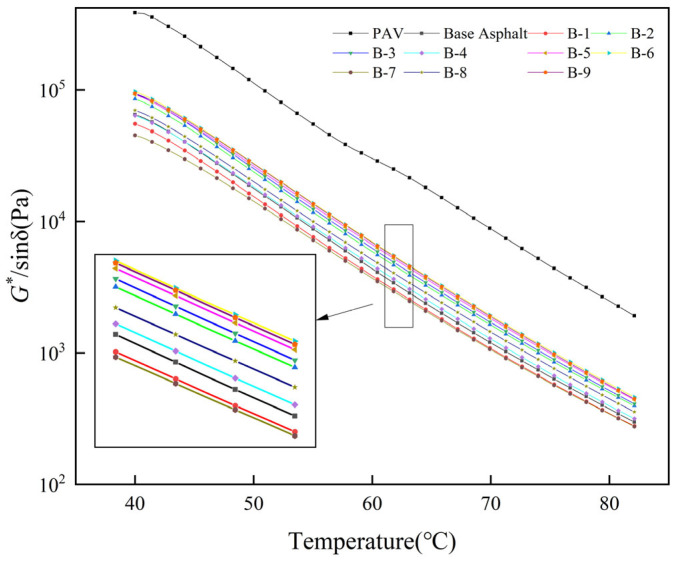
Rutting factors of different asphalts.

The rutting factor quantifies the resistance of asphalt pavements to deformation under load, with a higher rutting factor indicating enhanced high-temperature rutting resistance. Figure 5 illustrates the rutting factors of different asphalts, measured using the temperature scanning mode of the dynamic shear rheometer within the range of 40 °C to 82 °C.

Figure 5 demonstrates that the rutting factors of long-term aged asphalt, base asphalt, and the nine rejuvenated asphalts decrease with increasing temperature because elevated temperature intensifies molecular mobility inside the binder and reduces the elastic contribution [49]. B-1 and B-7, both with a heavy-component content of 50%, show lower rutting factors than the base asphalt, indicating that when the heavy-component content is relatively low, the softening and dilution effects of the light and medium components still dominate. The other seven rejuvenated asphalts, with heavy-component contents greater than 50%, show rutting factors that are higher than or close to that of the base asphalt, indicating that increasing the heavy fraction helps recover high-temperature deformation resistance. When the heavy-component content is 50–60%, the high-temperature performance of the rejuvenated asphalt is closest to that of the base asphalt, with B-4 being the closest and B-8 following. This is because the heavy fraction can supplement structural components reduced during asphalt aging, improve the solvation environment around asphaltene-rich domains, and strengthen the three-dimensional network structure [50], thereby enhancing high-temperature shear resistance. However, when the heavy-component content exceeds 60%, the marginal improvement in rutting resistance becomes limited, while low-temperature relaxation and construction workability may be adversely affected. Related studies also show that increased heavy fractions or asphaltene contents increase the penetration index (PI) and penetration–viscosity number (PVN), reduce temperature sensitivity, and make high-temperature rheological indicators change more slowly [51]. Therefore, a heavy-component range of 50–60% can be regarded as the preferred balance between structural support and excessive stiffening within the scope of this study: below this range, structural body is insufficient; above this range, high-temperature gains are limited and low-temperature risks increase.

This study applies the Christensen–Anderson–Marasteanu model to fit a master curve for the complex modulus, thereby describing the viscoelastic behavior of recycled asphalt over a wide spectrum of loading frequencies and temperatures. At lower loading frequencies (high temperature), a higher modulus typically improves the asphalt’s deformation resistance. In contrast, at higher loading frequencies (low temperature), an excessively high complex modulus may cause cracking under load.

**Figure 6:**
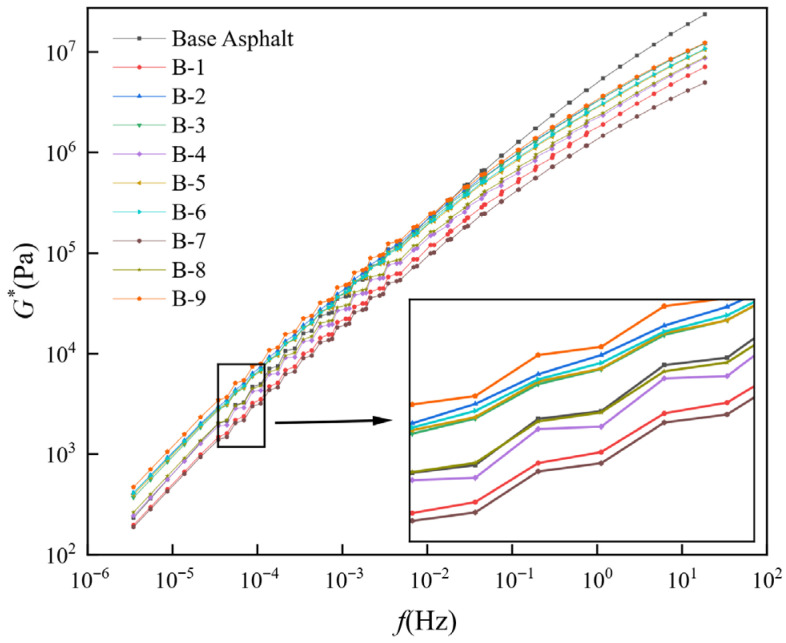
Master curve of the complex modulus of different asphalts.

As depicted in Figure 6, in the low-frequency region, the proportion of heavy components in B-1, B-4, B-7, and B-8 is below 60%, leading to a complex modulus of the recycled asphalt that is slightly lower than that of the base asphalt. In contrast, the proportion of heavy components in B-2, B-3, B-5, B-6, and B-9 exceeds 60%, leading to a higher complex modulus of the recycled asphalt than that of the base asphalt. In the high-frequency region, the complex modulus of all nine recycled asphalts increases at a slower rate and remains lower than that of base asphalt, indicating superior low-temperature crack resistance. Additionally, reducing the proportion of heavy components in the composite rejuvenator improves the low-temperature performance of recycled asphalt. To ensure sufficient high-temperature performance, the proportion of heavy components should remain between 50% and 60%.

#### 3.3.2. High-Temperature Creep Recovery Performance

**Figure 7:**
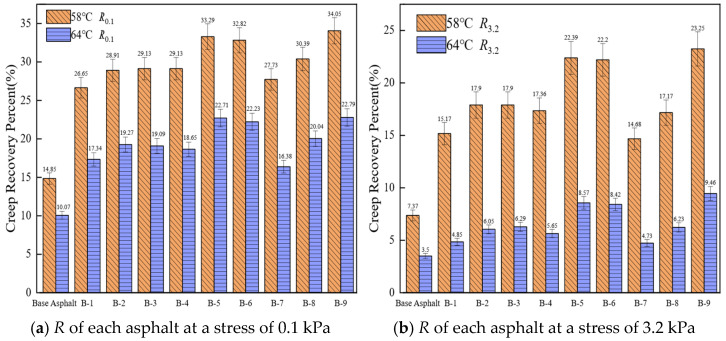
*R* of different asphalts under various temperatures and stresses.

The creep recovery rate (*R*) quantifies the ability of asphalt to recover from deformation after the load is removed. A higher *R* value indicates greater resistance to permanent deformation. Figure 7 illustrates *R* of both base asphalt and nine rejuvenated asphalt formulations under different stress levels.

As shown in Figure 7, the *R* value of asphalt decreases with increasing temperature and applied stress. The *R* values of the nine rejuvenated asphalts are 1 to 2 times greater than that of the base asphalt under different temperatures and applied stresses. This implies that rejuvenated asphalt demonstrates superior resistance to permanent deformation compared to base asphalt. Using B-7, B-8, and B-9 as examples, the rejuvenators in these formulations contain progressively higher proportions of heavy components. The *R* values of these three rejuvenated asphalts increase, indicating that higher proportions of heavy components in the rejuvenator reduce plastic deformation under load. For B-4, B-5, and B-6, as the proportion of heavy components increases and the medium component decreases, the *R* values first increase and then decrease. When the medium component in the rejuvenator constitutes 30%, the *R* value of the rejuvenated asphalt reaches its peak. This suggests that the proportion of medium components should be maintained around 30%; excessive amounts would reduce the system’s viscoelasticity, while insufficient amounts would impair the flexibility of the molecular chains, thus affecting the creep recovery rate. This is because methyl oleate effectively replenishes the light oils lost from aged asphalt due to oxidation and volatilization; however, it cannot repair the polymer molecules degraded by aging or restore the deteriorated asphalt polymer network [52].

Plastic deformation of asphalt under load is termed non-recoverable creep compliance (*J_nr_*). A lower *J_nr_* value indicates reduced susceptibility to rutting.

**Figure 8:**
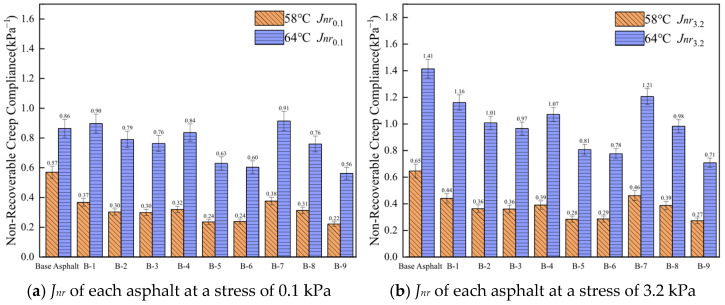
*J_nr_* of asphalt under different temperatures and stresses.

As shown in Figure 8, the *J_nr_* values of both base and rejuvenated asphalts are positively correlated with temperature and stress. Under identical testing temperature and stress conditions, the *J_nr_* values of all nine rejuvenated asphalts are lower than those of base asphalt, indicating that rejuvenated asphalt demonstrates superior resistance to permanent deformation compared to base asphalt. Additionally, at 58 °C, the *J_nr_* value of B-5 is the lowest among B-4, B-5, and B-6, further indicating that the medium component content should be maintained at approximately 30%. This result also shows that optimum performance does not depend solely on increasing the heavy fraction. Instead, the medium fraction provides compatibility and segmental mobility, the heavy fraction provides structural body, and the light fraction provides diffusion and initial softening, thereby achieving synergistic regulation.

The Performance Grade (PG) system evaluates the high-temperature stability and elastic recovery properties of asphalt. According to the ASTMD 7643-10 specification, the temperature corresponding to rutting factors |G*|/sin*δ* of 1.0 kPa for unaged asphalt and 2.2 kPa for short-term aged asphalt is determined via interpolation. The lower value is selected as the high-temperature grading temperature for the asphalt sample, as shown in Table 8.

As shown in Table 8, except for B-1 and B-7, the high-temperature grading of the other rejuvenated asphalts aligns with that of the base asphalt, indicating that these seven rejuvenators effectively restore the high-temperature grade of aged asphalt to match that of the base asphalt. When the rutting factor is 2.2 kPa, the failure temperatures of eight rejuvenated asphalts, excluding B-7, exceed that of the base asphalt, indicating that the rejuvenator composition improves the high-temperature performance of rejuvenated asphalt and effectively mitigates rutting damage. Table 8 also suggests that the rejuvenator’s heavy component content should be at least 50%, as lower levels would introduce excessive light components into the aged asphalt, diluting macromolecular substances and impairing the high-temperature performance of rejuvenated asphalt.

#### 3.3.3. Low-Temperature Rheological Properties

**Figure 9:**
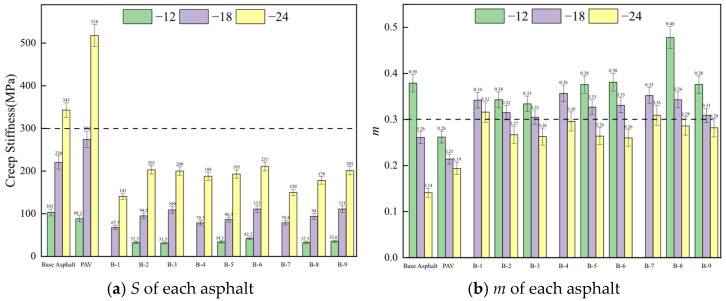
The low-temperature rheological parameters of different asphalts.

BBR is used to evaluate the stress relaxation and cracking resistance of asphalt binders under low-temperature conditions, reflecting their ability to release stress and resist cracking. This test determines the creep stiffness modulus (*S*) and creep rate (*m*) of asphalt under low-temperature conditions using a bending beam rheometer to assess its cracking resistance. Figure 9 displays the experimental results.

As shown in Figure 9, the *S* values of the nine rejuvenated asphalts are consistently lower than those of the base asphalt across all temperatures, while the *m* values are higher. A larger *m* value indicates superior stress relaxation performance of the asphalt [53]. Therefore, the nine rejuvenated asphalts exhibit superior low-temperature cracking resistance compared to the base asphalt. As shown in Figure 9b, at a test temperature of −24 °C, only B-1 and B-7 exhibit *m* values greater than 0.3, while the *m* values of the other seven rejuvenated asphalts are below 0.3. This indicates that increasing the proportion of light and medium components in the rejuvenator, while decreasing the proportion of heavy components, can improve the low-temperature cracking resistance of rejuvenated asphalt. This is because the heavy components mainly consist of stearic acid, arachidic acid, and tetracosanoic acid, all of which are long-chain saturated fatty acids. These fatty acids lack double bonds in their carbon chains, leading to a highly ordered molecular structure. This organized molecular arrangement allows the fatty acid molecules to pack closely, and as the temperature decreases, they are more likely to crystallize. This crystallization process leads to increased hardness and brittleness of the material [54,55].

#### 3.3.4. The Correlation Analysis of Thermal Oxidative Aging Resistance

The thermo-oxidative aging resistance of asphalt binders was assessed using the complex modulus (CAI) and phase angle (PAI) aging indices. A lower CAI together with a higher PAI corresponds to superior thermo-oxidative aging resistance.

**Figure 10:**
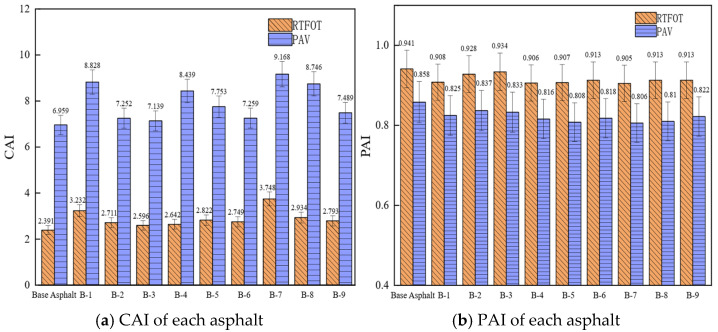
CAI and PAI of different asphalts.

As shown in Figure 10, after aging at 163 °C for 85 min and at 100 °C, 2.1 MPa for 20 h, the CAI of regenerated asphalt exceeds that of base asphalt. This indicates that small molecules with low thermal stability in the asphalt and rejuvenator volatilize, causing the aromatic and saturated components to aggregate with larger molecules, forming asphaltenes and resins. This increases the proportion of polar macromolecules in the asphalt, leading to a higher complex modulus of the regenerated asphalt and further performance degradation. After aging, the PAI of the asphalt falls below 1, indicating an increase in elastic components and a reduction in viscous components, leading to improved high-temperature performance and diminished low-temperature performance. Additionally, the PAI of the regenerated asphalt is lower than that of the base asphalt.

**Figure 11:**
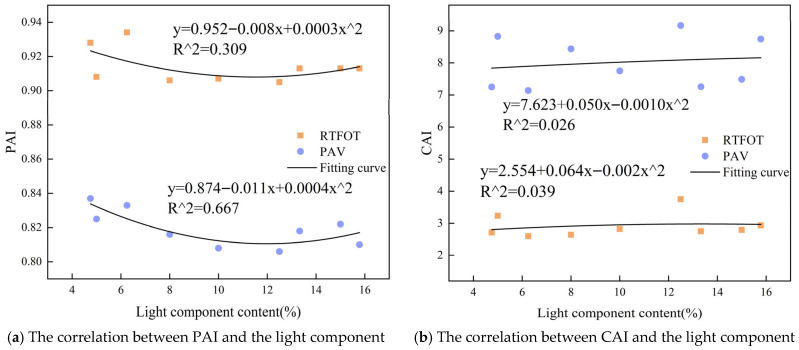

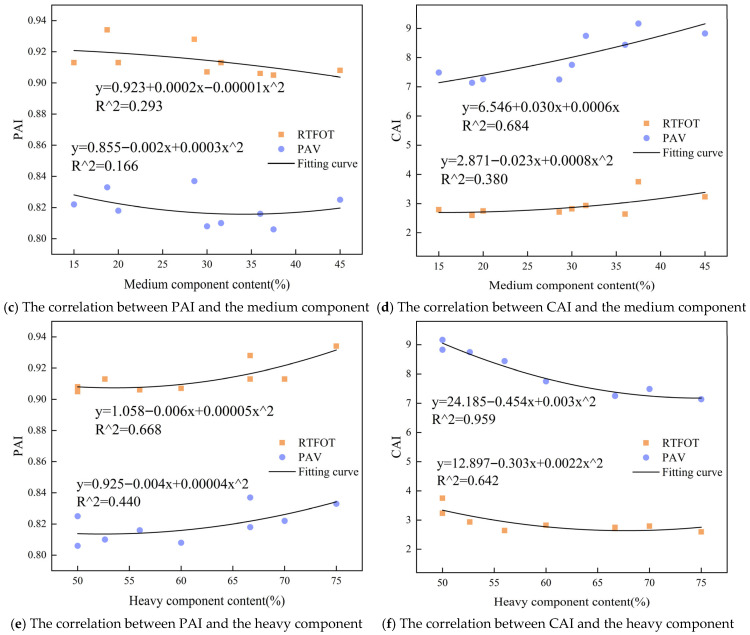
The correlation between PAI, CAI, and the light, medium, and heavy components.

To assess the influence of the light, medium, and heavy components of the composite rejuvenator on the thermal oxidative aging resistance of regenerated asphalt, the contents of these components from Table 7 were plotted along the *x*-axis, and the PAI and CAI of the regenerated asphalt were plotted along the *y*-axis. Scatter plots were created using Origin software 2024, as shown in Figure 11. Considering that the relationship between component proportion and aging index may be nonlinear and that the light, medium, and heavy components mutually constrain one another, a second-order polynomial fitting was adopted to describe possible curved trends. Compared with a first-order linear fit, a second-order fit can reflect potential inflection points or marginal effects in the variation in aging indices with component content; however, higher-order polynomials would risk overfitting because only nine formulations were tested. Therefore, Polynomial fitting was conducted using a second-order polynomial, and the coefficient of determination (R^2^) is shown in Table 9.

As shown in Table 9, the PAI during long-term aging correlates most strongly with the light component. Consequently, limiting volatilization of the light component reduces the change in the phase angle of rejuvenated asphalt. This is due to the relatively low temperature used in long-term aging, which preserves more light components. These components increase the proportion of viscous components in the aged asphalt, thus exerting a stronger influence on the phase angle; the CAI demonstrates the strongest correlation with the heavy component under both short- and long-term aging conditions. Under long-term aging conditions, the coefficient of determination (R^2^) between CAI and the heavy component is as high as 0.959, indicating an exceptionally strong linear correlation. Increasing the heavy component in the rejuvenator significantly improves the complex modulus of the rejuvenated asphalt and leads to minimal changes following aging. Overall, the findings indicate that increasing the heavy component content in the WCO rejuvenator is crucial for improving the long-term thermo-oxidative aging resistance of rejuvenated asphalt.

## 4. Conclusions

(1) Compositional regulation produces similar effects on the mass loss rate, saponification value, and 60 °C kinematic viscosity of the nine rejuvenators after heating. Increasing the light fraction with poor thermal stability makes the rejuvenator components more prone to volatilization at elevated temperatures, whereas the mass loss rate is inversely related to heavy-component content. The correlation analysis also shows that long-term aging PAI is most closely associated with the light component, whereas the R^2^ between long-term aging CAI and heavy-component content reaches 0.959, indicating that increasing the heavy fraction is a key factor for improving long-term thermo-oxidative aging resistance.
(2) The gas chromatography tests show that, in terms of chemical composition, the rejuvenators R-4 and R-8 exhibit compositional differences that align with the experimental expectations. Gel permeation chromatography analysis indicates that R-4 exhibits superior thermal oxidation stability relative to R-8. This is due to the higher content of small and medium-sized molecules in R-8, whereas R-4 has a higher concentration of larger molecules.
(3) The WCO fractions affect asphalt rheological properties through complementary rather than isolated functions. The light fraction promotes softening and low-temperature relaxation; the medium fraction maintains creep recovery capacity and molecular flexibility; and the heavy fraction provides structural strength and rutting resistance. When the heavy-component content is maintained at 50–60% and the medium-component content is approximately 30%, the rejuvenated asphalt achieves the closest balance between high- and low-temperature performance relative to the base asphalt. This composition range is based on the asphalt type and WCO source used in this study and should not be regarded as a universal fixed conclusion. Its main engineering value lies in proposing an adjustable design concept for fractionated WCO rejuvenators, which can reduce performance instability caused by crude WCO source variability and improve the economic and sustainable utilization of RAP.
(4) This study was limited to binder-level evaluation and did not cover aggregate–binder interactions, AFM-based characterization of chemical interactions and micro-morphological features, or the re-aging trajectory of asphalt pavement after 5–10 years of service. Future work should combine AFM, asphalt-mixture wheel tracking tests, and extended aging protocols to further verify micro-mechanisms and long-term durability of fractionated WCO rejuvenators.

## Figures and Tables

**Table 1:** Performance indexes of base asphalt.

Technical Indexes	Requirement	Test Result	Test Methods
Penetration (25 °C, 100 g, 5 s)	60–80	66.0	ASTM D5 [28]
Softening point (°C)	≥46	51.0	ASTM D36 [29]
Ductility (5 cm/min, 15 °C)	≥100	>100	ASTM D113 [30]
Viscosity (mPa·s,135 °C)	——	623.1	ASTM D4402 [31]
Flash point (°C)	≥260	292	ASTM D92 [32]
Density (g/cm^3^)	——	1.036	ASTM D1298 [33]
Mass loss after RTFOT (%)	±0.8	0.5	AASHTO T240 [34]

**Table 2:** Main compositional contents of the light, medium, and heavy components.

Technical Indexes	Test Result	Test Methods
Light component	methyl palmitate	200–300
Medium component	methyl oleate	300–400
Heavy component	long-chain fatty acids	500–1000

**Table 3:** Performance indicators of light, medium, and heavy components.

Technical Indexes	Light Component	Medium Component	Heavy Component	Test Methods
Density (g/cm^3^)	0.868	0.885	0.92	ASTM D4052 [35]
Dynamic viscosity at 60 °C (mm^2^/s)	9.347	10.392	36.214	ASTM D445 [36]
Iodine value (g/100 g)	68	97	116	ASTM D1510 [37]
Acid value (mgKOH/g)	0.7125	0.6349	10.623	ASTM D974 [38]
Moisture content (%)	0.03	0.04	0.09	ASTM D95 [39]

**Table 4:** Rejuvenators composition.

Rejuvenators Name	Light Component (g)	Medium Component (g)	Heavy Component (g)
R-1	5	45	50
R-2	5	30	70
R-3	5	15	60
R-4	10	45	70
R-5	10	30	60
R-6	10	15	50
R-7	15	45	60
R-8	15	30	50
R-9	15	15	70

**Table 5:** Chemical compositions of R-4 and R-8 rejuvenators.

Fatty Acid Names	Chemical Formula	R-4 (%)	R-8 (%)
Methyl Myristate	C_15_H_30_O_2_	4.78	4.59
Methyl Palmitate	C_16_H_34_O_2_	11.71	17.10
Methyl Linoleate	C_19_H_32_O_2_	4.34	3.82
Methyl Stearate	C_19_H_36_O_2_	9.97	9.52
Methyl Oleate	C_19_H_36_O_2_	40.60	33.51
Methyl Arachidate	C_21_H_42_O_2_	8.39	6.81
Methyl Erucate	C_23_H_44_O_2_	5.92	8.06
Methyl Behenate	C_23_H_46_O_2_	7.78	9.13
Methyl Lignocerate	C_25_H_50_O_2_	6.51	7.46

**Table 6:** Molecular weight distribution parameters of rejuvenators R-4 and R-8.

Molecular Weight	R-4	R-8
M_n_ (g/mol)	492	463
M_w_ (g/mol)	641	600
M_Z_ (g/mol)	943	918
PDI (M_w_/M_n_)	1.3	1.29

**Table 7:** Proportions of each rejuvenator component.

Rejuvenator Name	Light Component (%)	Medium Component (%)	Heavy Component (%)
B-1	5	45	50
B-2	4.75	28.58	66.67
B-3	6.25	18.75	75
B-4	8	36	56
B-5	10	30	60
B-6	13.33	20	66.67
B-7	12.5	37.5	50
B-8	15.78	31.58	52.64
B-9	15	15	70

**Table 8:** High-temperature PG grading of different asphalts.

Types of Asphalt	|G*|/sin*δ* = 1.0 kPa Failure Temperature (°C)	|G*|/sin*δ* = 2.2 kPa Failure Temperature (°C)	PG Grading Temperature (°C)	High-Temperature Grading
Base asphalt	71.47	71.95	71.47	PG70
B-1	69.10	72.31	69.10	PG64
B-2	73.98	75.98	73.98	PG70
B-3	74.37	76.04	74.37	PG70
B-4	72.00	75.70	72.39	PG70
B-5	74.94	77.46	74.97	PG70
B-6	75.37	77.60	75.37	PG70
B-7	68.90	71.50	68.90	PG64
B-8	72.39	75.67	72.00	PG70
B-9	75.18	78.31	75.18	PG70

**Table 9:** The R^2^ of the light, medium, and heavy components with PAI and CAI.

Component Names	R^2^ (PAI)		R^2^ (CAI)	
	Short-Term Aging	Long-Term Aging	Short-Term Aging	Long-Term Aging
Light component	0.309	0.667	0.039	0.026
Medium component	0.293	0.166	0.380	0.684
Heavy component	0.668	0.44	0.642	0.959

## Data Availability

Some or all data, models, or code that support the findings of this study are available from the corresponding author upon reasonable request.
